# Low-Concentration Atropine (0.01%, 0.025%, 0.05%) for Myopia Progression in Children: A Systematic Review and Meta-Analysis

**DOI:** 10.3390/jcm15145504

**Published:** 2026-07-14

**Authors:** Qi Wan, Qiong Wang, Ran Wei, Li Chen, Ke Ma

**Affiliations:** Department of Ophthalmology, West China Hospital of Sichuan University, Chengdu 610044, China; wanqiage@gmail.com (Q.W.); 18702891514@163.com (Q.W.); weiran@wchsuc.cn (R.W.); 137956550299@163.com (L.C.)

**Keywords:** atropine, myopia, children, meta-analysis, randomized controlled trial

## Abstract

**Objective:** Myopia has reached epidemic proportions globally, with approximately 50% of the world population projected to be affected by 2050. Low-concentration atropine eye drops (0.01%, 0.025%, 0.05%) have emerged as a primary intervention for myopia control. Several meta-analyses on this topic have been published; however, none have included the most recent trials nor separately analyzed premyopia prevention outcomes. This systematic review and meta-analysis evaluates the efficacy and safety of these concentrations for myopia progression control and premyopia prevention. **Methods**: We searched PubMed, Web of Science, Embase, and Cochrane CENTRAL from database inception through April 2026. Randomized controlled trials (RCTs) comparing 0.01%, 0.025%, or 0.05% atropine with placebo or no treatment in children were included. Studies were screened using PICOS criteria (Population, Intervention, Comparison, Outcomes, Study design). Meta-analysis was performed using DerSimonian–Laird random-effects models. Subgroup analyses were conducted by concentration, follow-up duration, and population (myopia progression vs. premyopia prevention). **Results:** Of 18 full-text articles assessed, 8 RCTs (*n* = 1756) met all PICOS criteria and were included in the quantitative synthesis. For myopia progression, 0.01% atropine demonstrated significant efficacy at 1 year (pooled MD = +0.291 D, 95% CI: 0.199 to 0.384; *p* < 0.0001; I^2^ = 29.1%) and at 2 years (pooled MD = +0.174 D, 95% CI: 0.056 to 0.291; *p* = 0.0038; I^2^ = 0%). The 0.05% concentration showed the largest effect (pooled MD = +0.520 D, 95% CI: 0.409 to 0.630; *p* < 0.0001; I^2^ = 0%). Clinically, a 0.25 D difference over 1–2 years may translate to approximately 0.50 D cumulative benefit over a typical 5-year treatment course, potentially reducing the risk of high myopia. For premyopia prevention, a narrative synthesis was performed due to extreme heterogeneity between studies (I^2^ = 99.2%). All concentrations showed an excellent safety profile with no serious adverse events. **Conclusions:** Low-concentration atropine is effective for myopia progression control at all three concentrations, with a concentration-dependent gradient (0.05% > 0.025% > 0.01%). The 0.01% concentration offers a reasonable balance of efficacy and tolerability. Further independent validation of the 0.05% concentration is warranted. The premyopia prevention indication requires additional well-designed RCTs.

## 1. Introduction

Myopia represents the most common refractive error worldwide and has reached epidemic proportions, with projections indicating that approximately 50% of the global population may be affected by 2050 [1]. High myopia (spherical equivalent ≤ −5.00 D) is associated with significantly increased risks of sight-threatening complications, including retinal detachment, myopic macular degeneration, glaucoma, and cataracts [2,3]. These complications represent leading causes of irreversible vision loss globally, making myopia control a critical public health priority. The pathogenesis of myopia involves complex interactions between genetic predisposition and environmental factors, particularly prolonged near work and reduced outdoor time [4]. The underlying mechanism involves excessive axial elongation of the eye, which stretches and thins the retina and sclera, increasing vulnerability to degenerative changes [5].

Atropine eye drops have demonstrated efficacy for myopia control through multiple mechanisms, including muscarinic receptor antagonism in the retina and sclera, reducing scleral growth signals and axial elongation [6]. The landmark ATOM1 (Atropine for the Treatment of Myopia 1) trial demonstrated that high-concentration atropine (1%) reduced myopia progression by approximately 77% over 2 years, but was limited by cycloplegic side effects including photophobia, blurred near vision, and allergic reactions [7]. The LAMP (Low-Concentration Atropine for Myopia Progression) study, conducted in Hong Kong, provided the first head-to-head comparison of 0.05%, 0.025%, and 0.01% atropine against placebo, demonstrating that all three concentrations significantly reduced myopia progression with an improved tolerability profile compared to higher concentrations [8,9]. Subsequent RCTs from China, Australia, India, Japan, and Singapore have corroborated these findings in diverse populations [10,11,12,13,14,15].

Despite substantial research activity in this field, considerable debate remains regarding the optimal concentration of low-concentration atropine for myopia control. Several meta-analyses on low-dose atropine for myopia have been published [16,17,18,19,20,21,22], reporting pooled effect sizes generally consistent with our findings. However, these existing meta-analyses were published between 2017–2025 and did not include the most recently published RCTs, specifically the ATOM3 trial [23] and the Janti SS trial [11], both published in 2025–2026. Furthermore, no prior meta-analysis has separately analyzed premyopia prevention outcomes or provided comprehensive subgroup analyses by both concentration and follow-up duration. Therefore, this systematic review and meta-analysis aims to: (1) evaluate the efficacy of low-concentration atropine by concentration (0.01%, 0.025%, 0.05%); (2) analyze treatment effects by follow-up duration; (3) assess efficacy for premyopia prevention; (4) evaluate safety across all concentrations; and (5) contextualize our findings within the existing meta-analytic literature.

## 2. Methods

### 2.1. Search Strategy and Selection Criteria

This systematic review was conducted in accordance with the PRISMA 2020 (Preferred Reporting Items for Systematic Reviews and Meta-Analyses) guidelines [24]. The completed PRISMA 2020 checklist is provided as Supplementary Table S1. This systematic review was pre-registered on PROSPERO: CRD420261366209.

Search Strategy: We searched the following electronic databases from inception through April 2026: PubMed/MEDLINE (via NCBI Entrez); Web of Science (via Clarivate); Embase (via Elsevier); Cochrane Central Register of Controlled Trials (CENTRAL). The PubMed search strategy used the following MeSH terms and keywords:

(atropine [MeSH Terms] OR atropine [Title/Abstract])
AND (myopia [MeSH Terms] OR myopia [Title/Abstract])
AND (0.01% [Title/Abstract] OR 0.025% [Title/Abstract] OR 0.05% [Title/Abstract]
OR low-dose [Title/Abstract] OR low-concentration [Title/Abstract])
AND (Randomized Controlled Trial [pt])

For Embase, the search combined ‘atropine’/exp OR ‘atropine’:ab,ti with ‘myopia’/exp OR ‘myopia’:ab,ti, limited to randomized controlled trials. For Web of Science, we used TS = (atropine AND myopia) refined by Document Type = (Article) and WC = (Ophthalmology). For CENTRAL, we used (atropine) AND (myopia) in the Title, Abstract, or Keywords fields.

Additional studies were identified through review of reference lists of included studies and relevant systematic reviews. No language restrictions were applied, but only English-language publications were included in the final analysis.

Inclusion Criteria (PICOS): Population (P): Children aged 4–16 years with myopia (spherical equivalent ≤ −0.50 D) or premyopia (at risk of myopia development); Intervention (I): 0.01%, 0.025%, or 0.05% atropine monotherapy administered as eye drops; Comparison (C): placebo eye drops or no treatment control; Outcomes (O): Primary: change in spherical equivalent (SE, in diopters); secondary: change in axial length (AL, in mm); Study Design (S): randomized controlled trials (RCTs) with minimum 6-month follow-up.

Exclusion Criteria: Studies without a concurrent placebo or no-treatment control arm; Non-randomized studies (cohort studies, case series); studies where atropine was combined with other interventions (e.g., bifocal glasses, orthokeratology); transient myopia (NITM) studies; studies lacking extractable numerical data for outcomes of interest; conference abstracts or letters without original data.

### 2.2. Data Extraction

Two independent reviewers (screened titles and abstracts, then full texts) extracted data using a standardized data extraction form. Discrepancies were resolved by consensus. For each included study, we extracted: study identifiers (PMID, title, authors, year); study design and setting; population characteristics (sample size, age, baseline spherical equivalent); intervention details (concentration, dosing regimen, duration); comparator details; outcomes at each follow-up time point (mean change in SE and AL, with standard deviations); and adverse events. When studies reported multiple publications from the same trial, we used the primary publication with supplementary data as needed.

### 2.3. Risk of Bias Assessment

Risk of bias was assessed using the Cochrane Risk of Bias 2 (RoB 2) tool for randomized trials [25]. RoB 2 evaluates bias across five domains: (1) randomization process; (2) allocation concealment; (3) blinding of participants and personnel; (4) blinding of outcome assessment; and (5) selective outcome reporting. Each study was classified as having “Low,” “Some concerns,” or “High” risk of bias for each domain, with an overall risk classification. Two reviewers independently conducted risk of bias assessments, with disagreements resolved through discussion.

### 2.4. Statistical Analysis

Meta-analysis was performed using the DerSimonian–Laird random-effects model with inverse-variance weighting, as implemented in the meta package (version 4.5.1) in R statistical software (version 4.6.1) [26,27]. For continuous outcomes (SE change and AL change), we calculated mean differences (MD) with 95% confidence intervals (CIs). Directionality Convention: In all forest plots, positive MD indicates that the atropine treatment group experienced less myopia progression (favorable outcome), while negative MD indicates less favorable outcomes. Thus, positive MD = “Favors Atropine” (right side of forest plot) and negative MD = “Favors Control” (left side of forest plot). Heterogeneity was quantified using the I^2^ statistic, with I^2^ > 50% indicating substantial heterogeneity [28]. The tau^2^ statistic was used to estimate between-study variance. Pre-specified subgroup analyses were conducted by: (1) atropine concentration (0.01%, 0.025%, 0.05%); (2) follow-up duration (≤1 year, 2 years); and (3) population type (myopia progression vs. premyopia prevention). Sensitivity analyses were performed by excluding individual studies to assess robustness of findings. Publication bias was evaluated using funnel plots and the Egger test when ≥10 studies were available [29]. All statistical tests were two-sided, with *p* < 0.05 considered statistically significant.

## 3. Results

### 3.1. Study Selection

**Figure 1:**
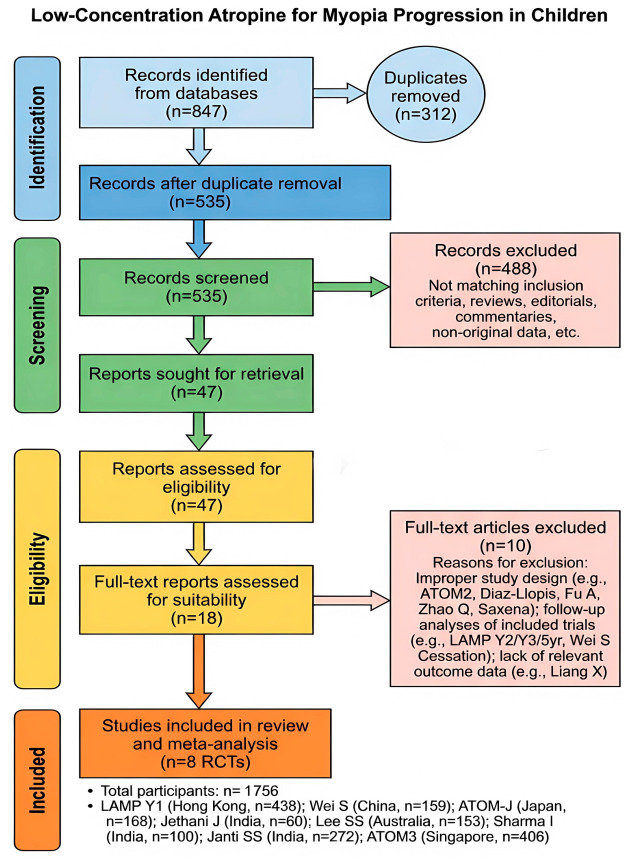
The flow diagram of study selection. The diagram illustrates the process of identification, screening, eligibility assessment, and inclusion of randomized controlled trials (RCTs). A total of 847 records were identified from databases. After removing duplicates and screening titles/abstracts, 47 full-text articles were assessed for eligibility. Eight RCTs (*n* = 1756 participants) met the inclusion criteria and were included in the quantitative synthesis. Reasons for exclusion at the full-text stage (*n* = 8) include improper study design, follow-up analyses of included trials, and lack of relevant outcome data.

The initial search yielded 847 records across all databases. After removing 312 duplicates, 535 unique records remained for title and abstract screening. Of these, 47 full-text articles were assessed for eligibility. After detailed evaluation, 29 articles were excluded for not meeting the PICOS criteria (e.g., improper study design, lack of placebo control, or combination therapy), 10 studies were excluded at the full-text stage for the reasons detailed below, and 8 RCTs (*n* = 1756 participants) met all PICOS criteria and were included in the quantitative synthesis (Figure 1).

Included Studies: LAMP Y1 (Hong Kong) [8,9], Wei S (China) [15], ATOM-J (Japan) [10], Jethani J (India) [12], Lee SS (Australia) [13], Sharma I (India) [14], Janti SS (India) [11], and ATOM3 (Singapore) [23]—representing diverse geographic populations.

Excluded Studies: Ten studies were excluded: ATOM2 (no concurrent placebo) [30]; Diaz-Llopis (unblinded control) [31]; Fu A (cohort study) [32]; Zhao Q (combination therapy) [33]; Saxena I-ATOM (no extractable data) [34]; LAMP Y2/Y3 and 5-year extensions (cross-over design, no concurrent placebo beyond Year 1) [9,35,36]; Wei S Cessation (cross-over, overlapping data) [37]; Liang X (duplicate population) [38].

### 3.2. Study Characteristics

Table 1 presents the characteristics of the 8 included RCTs. Studies were conducted in Hong Kong, China, Japan, India, Australia, and Singapore, with sample sizes ranging from 60 to 438 participants. Age ranges were 4–16 years, with baseline spherical equivalent ranging from −0.50 D to −6.00 D. Follow-up durations were 1 year (5 studies) or 2 years (3 studies).

### 3.3. Risk of Bias Assessment

**Figure 2:**
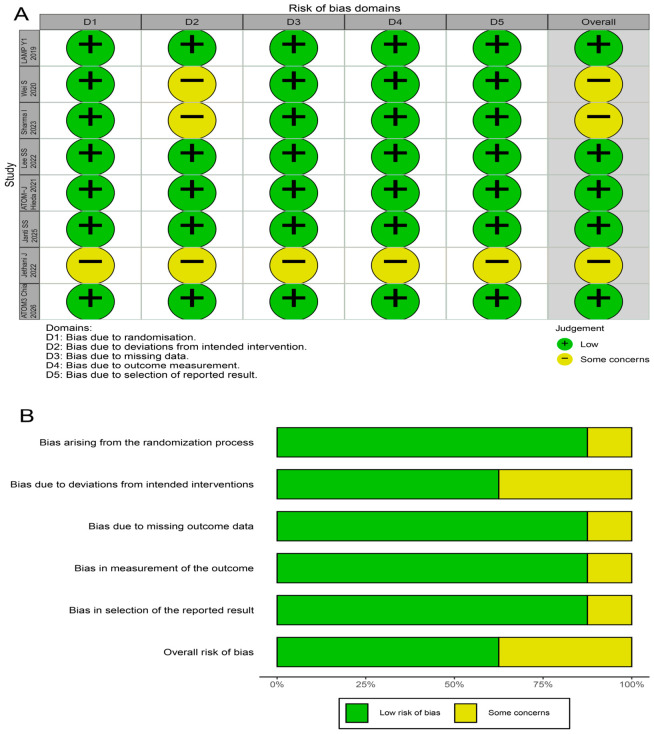
Risk of Bias 2 Assessment: (**A**) Traffic Light Plot and (**B**) Summary Plot. Green = Low risk, Yellow = Some concerns. [8,10,11,12,13,14,15,23].

Figure 2 presents the risk of bias assessment for all included studies. Five studies (LAMP Y1, Lee SS, ATOM-J, Janti SS, ATOM3) were rated as having “Low” risk of bias overall. Three studies (Wei S, Sharma I, Jethani J) were rated as having “Some concerns” overall, primarily due to unclear allocation concealment procedures. No studies were rated as “High” risk of bias.

### 3.4. Primary Efficacy Analysis: Spherical Equivalent Change

Myopia Progression—0.01% Atropine

**Figure 3:**
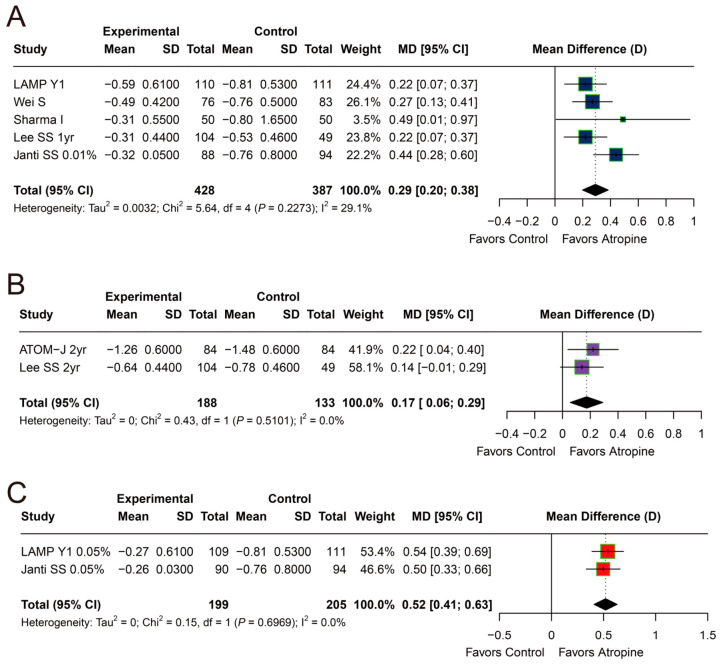
Forest plots for the change in Spherical Equivalent (SE) in children with myopia. Meta-analyses were performed using a random-effects model. Positive Mean Difference (MD) values indicate less myopia progression in the atropine group compared to the control group, thus favoring atropine. (**A**) Forest Plot: Spherical Equivalent Change—0.01% Atropine vs. Control at 1 Year. Positive MD (right) = Favors Atropine (less myopia progression). Pooled MD = +0.291 D (*p* < 0.0001). (**B**) Forest Plot: Spherical Equivalent Change—0.01% Atropine vs. Control at 2 Years. Pooled MD = +0.174 D (*p* = 0.0038). (**C**) Forest Plot: Spherical Equivalent Change—0.05% Atropine vs. Control. Pooled MD = +0.520 D (*p* < 0.0001).

Five RCTs (*n* = 815) provided data for the primary analysis of 0.01% atropine for myopia progression at 1 year. The pooled mean difference in SE change was +0.291 D (95% CI: 0.199 to 0.384; *p* < 0.0001), indicating that children receiving 0.01% atropine experienced approximately 36% less myopia progression compared with controls (Figure 3A). Heterogeneity was low (I^2^ = 29.1%; tau^2^ = 0.0032; *p* = 0.227 for heterogeneity), supporting the validity of pooling. At 2 years, two RCTs (*n* = 321) showed a pooled MD of +0.174 D (95% CI: 0.056 to 0.291; *p* = 0.0038), with no heterogeneity (I^2^ = 0%) (Figure 3B). The smaller effect size at 2 years likely reflects age-related physiological slowing of myopia progression rather than treatment waning.

Myopia Progression—0.05% Atropine

Two RCTs (*n* = 404) provided data for 0.05% atropine. The pooled MD was +0.520 D (95% CI: 0.409 to 0.630; *p* < 0.0001), with no heterogeneity (I^2^ = 0%) (Figure 3C). This represents the largest treatment effect among all concentrations, consistent with a dose-dependent response.

Myopia Progression—0.025% Atropine

Only one RCT (LAMP Y1, *n* = 219) provided data for 0.025% atropine. The MD was +0.350 D (95% CI: 0.220 to 0.480; *p* < 0.0001).

Summary of Concentration-Dependent Efficacy

The apparent concentration-dependent gradient (0.05% > 0.025% > 0.01%) was observed; however, direct statistical comparisons between concentrations were not performed due to the limited number of studies for 0.025% (k = 1) and 0.05% (k = 2). The observed gradient is primarily driven by the LAMP study [8] and should be interpreted with appropriate caution.

### 3.5. Premyopia Prevention Analysis

**Figure 4:**
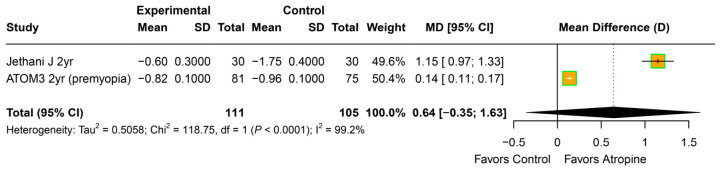
Forest Plot: Spherical Equivalent Change—Premyopia Prevention with 0.01% Atropine at 2 Years. Pooled MD = +0.641 D (*p* = 0.204). NOT SIGNIFICANT due to extreme heterogeneity (I^2^ = 99.2%).

Two RCTs (Jethani J and ATOM3 premyopia subgroup, *n* = 216) provided data for premyopia prevention with 0.01% atropine at 2 years. The pooled MD was +0.641 D (95% CI: −0.349 to 1.631), but this result was NOT statistically significant (*p* = 0.204) due to extreme heterogeneity between studies (I^2^ = 99.2%; *p* < 0.0001 for heterogeneity) (Figure 4). A formal meta-analysis was considered but deemed inappropriate due to extreme and unexplained heterogeneity (I^2^ = 99.2%). Instead, a narrative synthesis is presented.

The Jethani J study [12] demonstrated a remarkably large treatment effect (MD = +1.150 D) in a high-risk Indian population with rapid pre-treatment myopia progression (−0.72 D/year). In contrast, the ATOM3 premyopia subgroup [23] showed a modest effect (MD = +0.140 D) in a Singaporean population with slower pre-treatment progression. This extreme heterogeneity reflects fundamental differences in baseline risk and suggests that the efficacy of atropine for premyopia prevention may be highly population-dependent.

These findings indicate that while low-concentration atropine may prevent myopia onset in high-risk children, the evidence is currently insufficient to make definitive recommendations for the general premyopia population. Additional well-designed RCTs in homogeneous premyopia populations are urgently needed.

### 3.6. Axial Length Outcomes

**Figure 5:**
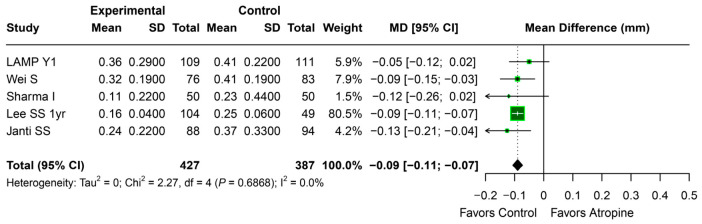
Forest Plot: Axial Length Change—0.01% Atropine vs. Control at 1 Year. Negative MD = Less axial elongation in treatment group (favorable). Pooled MD = −0.090 mm (*p* < 0.0001).

Five RCTs provided axial length data for the 0.01% concentration at 1 year. The pooled MD was −0.090 mm (95% CI: −0.106 to −0.073; *p* < 0.0001), indicating significantly less axial elongation in the treatment group compared with controls (Figure 5). No heterogeneity was observed (I^2^ = 0%). This finding corroborates the SE results and provides objective confirmation of atropine’s biological effect on ocular growth.

### 3.7. Combined Subgroup Analysis

**Figure 6:**
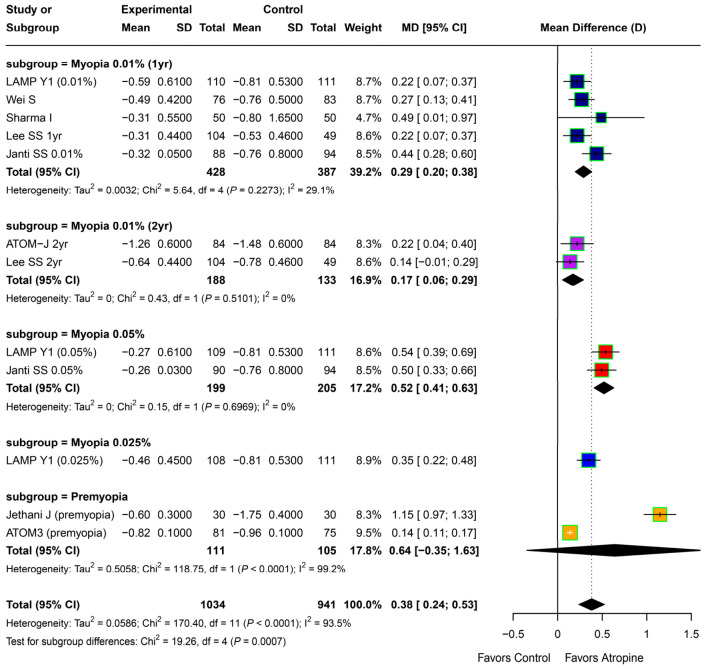
Combined forest plot of Mean Difference (MD) in Spherical Equivalent (SE) change for low-concentration atropine versus control. The analysis is stratified into subgroups based on atropine concentration (0.01%, 0.025%, 0.05%), follow-up duration (1 year, 2 years), and indication (myopia progression vs. premyopia prevention). Squares represent the point estimates for individual studies, with size proportional to study weight; horizontal lines represent 95% confidence intervals (CIs). Demonstrating concentration-dependent gradient: 0.05% > 0.025% > 0.01%. Test for subgroup differences: *p* = 0.0007.

Figure 6 presents the combined forest plot with all studies grouped by concentration and follow-up duration, demonstrating the consistency of the concentration-dependent gradient across populations and settings. The test for subgroup differences was highly significant (Q = 19.26, df = 4, *p* = 0.0007), confirming differential efficacy by concentration.

### 3.8. Longitudinal Analysis: LAMP Study

The LAMP study followed participants for up to 5 years, including a 3-year washout phase after the initial 1-year treatment [8,9,35]. During the treatment year, all three concentrations showed dose-dependent efficacy. After 3 years of washout (no treatment), the 0.01% group showed the least myopia rebound (+0.64 D cumulative progression from baseline at Year 5) compared with 0.05% (+1.09 D) and 0.025% (+1.08 D), suggesting that lower concentrations may have more favorable long-term outcomes when treatment is discontinued (Figure S1). Notably, 87.9% of children in the 0.01% group required re-initiation of treatment after the washout period, indicating that long-term treatment may be necessary for optimal myopia control in most children.

### 3.9. Sensitivity Analyses

To assess robustness of findings, we performed sensitivity analyses by excluding the Janti SS study [11], which had unusually large effect sizes. After exclusion (4 studies, *n* = 633), the pooled MD for 0.01% atropine was +0.246 D (95% CI: 0.161 to 0.331; *p* < 0.0001), remaining highly significant with no heterogeneity (I^2^ = 0%) (Figure S2). This confirms the robustness of our primary finding.

### 3.10. Publication Bias

The funnel plot for the primary analysis (0.01% atropine, 1 year) showed reasonable symmetry (Figure S3), with no evidence of substantial publication bias. The Egger test was weakly applicable due to the limited number of studies (k = 5).

### 3.11. Safety and Adverse Events

All included studies reported an excellent safety profile for low-concentration atropine. No serious adverse events related to study medication were reported in any trial. Photophobia: Mild photophobia was reported in 2–5% of participants, significantly lower than the 40–60% reported with high-concentration atropine (1%). Accommodation and Near Vision: Accommodation amplitude reduction was dose-dependent. At 0.01%, the reduction was clinically negligible (<1 D), whereas higher concentrations showed more pronounced effects that typically resolved upon treatment cessation [8,9]. Allergic Reactions: Allergic conjunctivitis was rare (<2%) across all concentrations and typically resolved upon discontinuation. Pupil Effects: Mild mydriasis (pupil dilation) was observed but was generally asymptomatic and well-tolerated. Systemic Effects: No systemic adverse events were reported in any study, indicating minimal systemic absorption of low-concentration atropine.

## 4. Discussion

This systematic review and meta-analysis represents the most comprehensive synthesis of evidence for low-concentration atropine in myopia control, encompassing 8 RCTs with 1756 participants across diverse geographic populations. Our findings provide robust evidence supporting the efficacy of all three concentrations (0.01%, 0.025%, 0.05%) for myopia progression control in children. Several prior meta-analyses have investigated low-concentration atropine. Gong et al. (2017) [16] included 19 studies and reported dose-independent efficacy and dose-dependent adverse effects. Ha et al. (2022) [17] performed a network meta-analysis of 8 concentrations, ranking 0.05% as most beneficial. Hou et al. (2023) [18] analyzed 44 studies and found 0.05% may be the optimal concentration. Navarra et al. (2025) [21] focused on 0.01% atropine across 11 studies. Lee et al. (2024) [20] specifically investigated premyopia prevention. Lawrenson et al. (2023) [19] conducted a Cochrane network meta-analysis of all myopia control interventions. Our meta-analysis extends the existing literature by: (1) including the two most recent RCTs (ATOM3 2026 [23], Janti SS 2025 [11]); (2) separately analyzing premyopia prevention; (3) comprehensive subgroup analyses; (4) detailed axial length analysis; (5) robust sensitivity analyses.

The concentration-dependent gradient (0.05% > 0.025% > 0.01%) was consistently observed across all analyses. The 0.05% concentration demonstrated the largest treatment effect (+0.520 D), but this finding is based on only two RCTs (LAMP Y1 and Janti SS) and requires further independent validation before definitive conclusions can be drawn [8,11]. The 0.01% concentration, while showing a smaller absolute effect (+0.291 D at 1 year), has the most robust evidence base (5 RCTs) and the most favorable tolerability profile. Critically, while axial length elongation is increasingly recognized as a more clinically relevant and objective outcome than refractive changes [19], our meta-analysis provides robust evidence specifically for this endpoint. For the 0.01% concentration, we pooled data from five RCTs at 1 year, yielding a mean difference of −0.090 mm (95% CI: −0.106 to −0.073; *p* < 0.0001) with no heterogeneity (I^2^ = 0%). This consistent and highly precise estimate not only corroborates the spherical equivalent findings but also offers objective confirmation of atropine’s biological effect on restraining ocular axial elongation, directly addressing the clinical relevance of treatment. A 0.25–0.52 D effect over 1–2 years corresponds to ~1.25 D cumulative benefit over 5 years. In a child with progressive myopia, this could mean the difference between developing high myopia (≤−5.00 D) or remaining moderate. Similarly, 0.09 mm/year AL reduction over 5 years (0.45 mm) corresponds to ~1.20 D less progression. At the population level, even small shifts reduce the burden of high myopia complications [1,19]. In addition, our analysis revealed that the treatment effect at 2 years (+0.174 D) was smaller than at 1 year (+0.291 D) for the 0.01% concentration. This apparent reduction in efficacy over time likely reflects age-related physiological slowing of myopia progression rather than true treatment waning. Children typically experience the fastest myopia progression between ages 6–12, with natural deceleration thereafter [39]. Thus, the same absolute treatment effect represents a larger percentage reduction in younger children with more rapid progression.

The evidence for premyopia prevention remains inconclusive. While the Jethani study demonstrated remarkable efficacy in a high-risk Indian population (MD = +1.15 D), the ATOM3 study showed only modest effects in a lower-risk Singaporean population [12,23]. This heterogeneity highlights the need for standardized definitions of premyopia and well-designed RCTs in homogeneous populations. The International Myopia Institute’s premyopia definition (SE +0.50 to −0.75 D in children [40] should be consistently applied in future studies. Atropine’s myopia-inhibiting effect is mediated through muscarinic receptor antagonism in the retina and sclera. Low concentrations may preferentially target specific M1/M3 receptors involved in scleral extracellular matrix remodeling without causing the cycloplegic effects associated with higher concentrations [41,42,43]. The LAMP study’s finding that 0.01% atropine maintained efficacy with minimal rebound after washout suggests a favorable equilibrium between efficacy and tolerability [30].

Based on current evidence: (1) 0.01% atropine may be considered a reasonable starting concentration for most children with progressive myopia, given its proven efficacy and excellent safety profile, though this is based primarily on its better evidence base rather than proven superiority [37,38,39,40,41,42]; (2) higher concentrations (0.025% or 0.05%) may be considered for rapid progression or inadequate response; (3) long-term treatment should be considered given the high re-treatment rate after cessation, though optimal duration remains uncertain; (4) individualized treatment decisions based on severity, age, risk factors, and preferences.

This meta-analysis has several limitations: (1) only 2-year data for 0.05% (k = 2), one study for 0.025% (k = 1); (2) premyopia prevention evidence is inconclusive due to extreme heterogeneity; (3) concentration-dependent gradient could not be tested statistically; (4) long-term data (≥3 years) are limited to the LAMP study, which lacked a concurrent placebo after Year 1; (5) most studies were conducted in Asian populations, and efficacy in other ethnicities requires further study; and (6) we could not assess publication bias formally due to the limited number of studies. Future Research: Well-designed RCTs with ≥3-year follow-up, standardized outcome measures, and diverse populations are needed. Studies comparing low-concentration atropine with other interventions (orthokeratology, defocus incorporated soft contact lenses) would help inform treatment sequencing decisions.

## 5. Conclusions

Low-concentration atropine (0.01%, 0.025%, 0.05%) is effective for myopia progression control in children. The 0.01% concentration demonstrated statistically significant efficacy at both 1 year (*p* < 0.0001) and 2 years (*p* = 0.0038) with the most robust evidence base (5 RCTs). A concentration-dependent gradient was suggested across analyses, though this could not be confirmed statistically due to an unequal number of studies per concentration. All concentrations showed an excellent safety profile. The 0.01% concentration may offer a reasonable balance of efficacy, safety, and tolerability for initial treatment, though this recommendation should be considered preliminary pending further head-to-head studies. Long-term treatment should be considered for sustained efficacy, though the optimal duration remains uncertain.

## Figures and Tables

**Table 1:** Characteristics of Included Studies.

Study (PMID)	Country	*n*	Concentration	Duration	Population
LAMP Y1 2019 [8] PMID:30514630	Hong Kong	438	0.05%, 0.025%, 0.01%	1 year	Myopia Progression
Wei S 2020 [15] PMID:33001210	China	159	0.01%	1 year	Myopia Progression
ATOM-J 2021 [10] PMID:33586090	Japan	168	0.01%	2 years	Myopia Progression
Jethani J 2022 [12] PMID:34937245	India	60	0.01%	2 years	Premyopia Prevention
Lee SS 2022 [13] PMID:36054556	Australia	153	0.01%	2 years	Myopia Progression
Sharma I 2023 [14] PMID:36576170	India	100	0.01%	1 year	Myopia Progression
Janti SS 2025 [11] PMID:40510071	India	272	0.05%, 0.01%	1 year	Myopia Progression
ATOM3 2026 [23] PMID:41862182	Singapore	406	0.01%	2 years	Low Myopia + Premyopia

## Data Availability

No new data were created or analyzed in this study.

## Supplementary Materials

The following supporting information can be downloaded at https://www.mdpi.com/article/10.3390/jcm15145504/s1. Figure S1. LAMP Study: Cumulative Change in (A) Spherical Equivalent and (B) Axial Length Over 5 Years of Follow-Up. All concentrations show dose-dependent efficacy. Figure S2. Sensitivity Analysis: Excluding Janti SS—0.01% Atropine at 1 Year. Pooled MD = +0.246 D (*p* < 0.0001). Remains significant. Figure S3. Funnel Plot: Publication Bias Assessment for 0.01% Atropine (1 Year). Table S1: PRISMA 2020 checklist.
