# Supplementary material for: Low-Concentration Atropine (0.01%, 0.025%, 0.05%) for Myopia Progression in Children: A Systematic Review and Meta-Analysis
Source: J Clin Med. 2026 Jul 14;15(14):5504. doi: 10.3390/jcm15145504 (PMC13413086; doi:10.3390/jcm15145504)
Supplement: Supplementary file 1 [file jcm-15-05504-s001.zip › supplementary-prisma checklist.pdf]

PRISMA 2020 Checklist

Section and Topic	Item #	Checklist item	Location where item is reported
**TITLE**			
Title	1	Identify the report as a systematic review.	Title page, line 1: "Low-Concentration Atropine (0.01%, 0.025%, 0.05%) for Myopia Progression in Children: A Systematic Review and Meta-Analysis"
**ABSTRACT**			
Abstract	2	See the PRISMA 2020 for Abstracts checklist.	Abstract, lines [6]–[11] (Objective, Methods, Results, Conclusions, Keywords) — contains all PRISMA abstract elements
**INTRODUCTION**			
Rationale	3	Describe the rationale for the review in the context of existing knowledge.	Introduction, paragraphs [12]–[15]: Global myopia prevalence (ref[1]), high myopia complications (ref[2-5]), atropine pharmacology (ref[6]), ATOM1/LAMP trials (ref[7-10]), gap in comprehensive meta-analysis
Objectives	4	Provide an explicit statement of the objective(s) or question(s) the review addresses.	Introduction, paragraph [15], final sentence: "no comprehensive meta-analysis has yet synthesized the totality of evidence across all low-concentration atropine RCTs with quantitative pooling of both refractive and axial length outcomes"
**METHODS**			

PRISMA 2020 Checklist

Section and Topic	Item #	Checklist item	Location where item is reported
Eligibility criteria	5	Specify the inclusion and exclusion criteria for the review and how studies were grouped for the syntheses.	Methods, lines [26]–[27]: PICOS — P: children 4–16y with myopia (SE ≤ −0.50 D) or premyopia; I: 0.01%, 0.025%, 0.05% atropine monotherapy; C: placebo/no treatment; O: SE change (primary), AL change (secondary); S: RCTs with ≥6-month follow-up. Studies grouped by concentration, follow-up duration, and indication (myopia progression vs premyopia prevention).
Information sources	6	Specify all databases, registers, websites, organisations, reference lists and other sources searched or consulted to identify studies. Specify the date when each source was last searched or consulted.	Methods, lines [19]–[25]: PubMed/MEDLINE (via NCBI Entrez), Web of Science (via Clarivate), Embase (via Elsevier), Cochrane CENTRAL. Searched from database inception through April 2026. No language restrictions applied (English-language publications only in final analysis). Supplementary review of reference lists.
Search strategy	7	Present the full search strategies for all databases, registers and websites, including any filters and limits used.	Methods, lines [20]–[24]: Full PubMed strategy provided: (atropine[MeSH Terms] OR atropine[Title/Abstract])

Section and Topic	Item #	Checklist item	Location where item is reported
			AND (myopia[MeSH Terms] OR myopia[Title/Abstract]) AND (0.01% OR 0.025% OR 0.05% OR low-dose OR low-concentration) AND (Randomized Controlled Trial[pt])
Selection process	8	Specify the methods used to decide whether a study met the inclusion criteria of the review, including how many reviewers screened each record and each report retrieved, whether they worked independently, and if applicable, details of automation tools used in the process.	Methods, line [29]: "Two independent reviewers (screened titles and abstracts, then full texts)" and "Discrepancies were resolved by consensus"
Data collection process	9	Specify the methods used to collect data from reports, including how many reviewers collected data from each report, whether they worked independently, any processes for obtaining or confirming data from study investigators, and if applicable, details of automation tools used in the process.	Methods, lines [29]: "Two independent reviewers extracted data using a standardized data extraction form." Discrepancies resolved by consensus. Corresponding authors not contacted for additional data.
Data items	10a	List and define all outcomes for which data were sought. Specify whether all results that were compatible with each outcome domain in each study were sought (e.g. for all measures, time points, analyses), and if not, the methods used to decide which results to collect.	Methods, line [26] and Results lines [107]–[127]: Primary outcome (SE change in D); secondary (AL change in mm). All time points sought (1-year, 2-year). All results within each domain collected.
	10b	List and define all other variables for which data were sought (e.g. participant and intervention characteristics, funding sources). Describe any assumptions made about any missing or unclear information.	Methods, lines [29]: Extracted variables: study identifiers (PMID, title, authors, year),

Section and Topic	Item #	Checklist item	Location where item is reported
			design and setting, population (sample size, age, baseline SE), intervention (concentration, dosing regimen, duration), comparator, outcomes at each follow-up point (mean change ± SD), adverse events.
Study risk of bias assessment	11	Specify the methods used to assess risk of bias in the included studies, including details of the tool(s) used, how many reviewers assessed each study and whether they worked independently, and if applicable, details of automation tools used in the process.	Methods, line [31]: Cochrane Risk of Bias 2 (RoB 2) tool across five domains. Two reviewers independently assessed each study.
Effect measures	12	Specify for each outcome the effect measure(s) (e.g. risk ratio, mean difference) used in the synthesis or presentation of results.	Methods, line [33]: Mean difference (MD) with 95% CIs for continuous outcomes (SE change, AL change). DerSimonian-Laird random-effects model, inverse-variance weighting.
Synthesis methods	13a	Describe the processes used to decide which studies were eligible for each synthesis (e.g. tabulating the study intervention characteristics and comparing against the planned groups for each synthesis (item #5)).	Methods, lines [26]–[27]: Studies grouped for synthesis by: (1) atropine concentration (0.01%, 0.025%, 0.05%), (2) follow-up duration (1-year, 2-year), (3) indication (myopia progression, premyopia prevention). Only RCTs meeting PICOS criteria eligible.
	13b	Describe any methods required to prepare the data for presentation or synthesis, such as handling of missing summary statistics, or data conversions.	Methods, line [33]: SE change and AL change

PRISMA

PRISMA 2020 Checklist

Section and Topic	Item #	Checklist item	Location where item is reported
			extracted as mean ± SD. Directionality convention: positive MD = favors atropine (less myopia progression). No data conversions were required for included studies.
	13c	Describe any methods used to tabulate or visually display results of individual studies and syntheses.	Methods, line [33] and Results: Forest plots (Figures 3–6, Figures S1–S2) for all meta-analyzed outcomes. Combined forest plot with subgroup stratification (Figure 6). Box plots for longitudinal LAMP data.
	13d	Describe any methods used to synthesize results and provide a rationale for the choice(s). If meta-analysis was performed, describe the model(s), method(s) to identify the presence and extent of statistical heterogeneity, and software package(s) used.	Methods, line [33]: DerSimonian-Laird random-effects model; I^2 statistic, Cochran's Q, tau^2 (REML) for heterogeneity. Software: R, meta package (v8.3-0). Directionality: positive MD = "Favors Atropine".
	13e	Describe any methods used to explore possible causes of heterogeneity among study results (e.g. subgroup analysis, meta-regression).	Methods and Results, line [131] (Section "Sensitivity Analyses"): Exclusion of Janti SS (10.7759/cureus.84010) due to unusually large effect sizes.
	13f	Describe any sensitivity analyses conducted to assess robustness of the synthesized results.	Methods, line [131] and Results, lines [130]–[131]: Sensitivity analysis by excluding

PRISMA 2020 Checklist

Section and Topic	Item #	Checklist item	Location where item is reported
			Janti SS study (Figure S2). Results remain significant (pooled MD = +0.246 D, P < 0.0001, I^2 = 0%).
Reporting bias assessment	14	Describe any methods used to assess risk of bias due to missing results in a synthesis (arising from reporting biases).	Methods, lines [133]: Funnel plot for primary analysis (0.01% atropine, 1 year). Egger's test noted as not applicable (k = 5). No trim-and-fill or Begg's test described.
Certainty assessment	15	Describe any methods used to assess certainty (or confidence) in the body of evidence for an outcome.	Methods, line [33]: No formal GRADE assessment. Heterogeneity evaluated via I^2 and Cochran's Q. Sensitivity analysis and subgroup analysis conducted.
**RESULTS**			
Study selection	16a	Describe the results of the search and selection process, from the number of records identified in the search to the number of studies included in the review, ideally using a flow diagram.	Results, lines [36]–[39] and Figure 1: 847 records identified; 312 duplicates removed; 535 screened; 47 full-text assessed; 18 reviewed in detail; 8 RCTs (n = 1,756) included. PRISMA 2020 flow diagram in Figure 1.
	16b	Cite studies that might appear to meet the inclusion criteria, but which were excluded, and explain why they were excluded.	Results, line [38]: 10 studies excluded with reasons: ATOM2 (no concurrent placebo); Diaz-Llopis (unblinded control); Fu A (cohort);

PRISMA

PRISMA 2020 Checklist

Section and Topic	Item #	Checklist item	Location where item is reported
			Zhao Q (combination therapy); Saxena I-ATOM (no extractable data); LAMP Y2/Y3/5-year (cross-over, no concurrent placebo); Wei S Cessation (cross-over, overlapping data); Liang X (duplicate population).
Study characteristics	17	Cite each included study and present its characteristics.	Results, lines [40]–[103] and Table 1: Characteristics of 8 included RCTs: LAMP Y1 (Hong Kong), Wei S (China), ATOM-J (Japan), Jethani J (India), Lee SS (Australia), Sharma I (India), Janti SS (India), ATOM3 (Singapore). Sample sizes 60–438, age 4–16 years, SE −0.50 to −6.00 D.
Risk of bias in studies	18	Present assessments of risk of bias for each included study.	Results, lines [104]–[106] and Figure 2: RoB 2 assessment traffic light plot and summary plot. Five studies (LAMP Y1, Lee SS, ATOM-J, Janti SS, ATOM3): Low risk. Three studies (Wei S, Sharma I, Jethani J): Some concerns (unclear allocation concealment). No studies rated High risk.
Results of individual studies	19	For all outcomes, present, for each study: (a) summary statistics for each group (where appropriate) and (b) an effect estimate and its precision (e.g. confidence/credible interval), ideally using structured tables or plots.	Results, lines [107]–[127] (Figures 3–6):

Section and Topic	Item #	Checklist item	Location where item is reported
			Study-level summary statistics and pooled effect estimates (MD, 95% CI) for each outcome. Forest plots display individual study weights, point estimates, and confidence intervals.
Results of syntheses	20a	For each synthesis, briefly summarise the characteristics and risk of bias among contributing studies.	Results, lines [104]–[106] (Figure 2) and [107]–[127]: Risk of bias of contributing studies summarized in Figure 2. Study characteristics in Table 1.
	20b	Present results of all statistical syntheses conducted. If meta-analysis was done, present for each the summary estimate and its precision (e.g. confidence/credible interval) and measures of statistical heterogeneity. If comparing groups, describe the direction of the effect.	Results, lines [107]–[127]: Pooled MD with 95% CI, P-values, I^2 for each synthesis. 0.01% at 1y: MD = +0.291 D ($P < 0.0001$, $I^2 = 29.1\%$); 0.01% at 2y: MD = +0.174 D ($P = 0.0038$, $I^2 = 0\%$); 0.05%: MD = +0.520 D ($P < 0.0001$, $I^2 = 0\%$); premyopia: MD = +0.641 D ($P = 0.204$, $I^2 = 99.2\%$).
	20c	Present results of all investigations of possible causes of heterogeneity among study results.	Results, lines [109]–[127]: I^2 and Cochran's Q reported; 0.01% at 1y $I^2 = 29.1\%$ (low); 2y $I^2 = 0\%$; 0.05% $I^2 = 0\%$; premyopia $I^2 = 99.2\%$ (extreme). Heterogeneity sources discussed: Janti SS large effect; premyopia

Section and Topic	Item #	Checklist item	Location where item is reported
			population differences.
	20d	Present results of all sensitivity analyses conducted to assess the robustness of the synthesized results.	Results, lines [130]–[131], Figure S2: Sensitivity analysis excluding Janti SS. Pooled MD remains significant: +0.246 D (95% CI: 0.161–0.331, $P < 0.0001$), $I^2 = 0\%$.
Reporting biases	21	Present assessments of risk of bias due to missing results (arising from reporting biases) for each synthesis assessed.	Results, line [133] and Figure S: Funnel plot for primary analysis shows reasonable symmetry. Egger's test not applicable ($k = 5$). No evidence of substantial publication bias.
Certainty of evidence	22	Present assessments of certainty (or confidence) in the body of evidence for each outcome assessed.	Results, lines [107]–[135]: I^2 values, sensitivity analysis results, and publication bias assessment reported. No formal GRADE certainty assessment.
**DISCUSSION**			
Discussion	23a	Provide a general interpretation of the results in the context of other evidence.	Discussion, lines [136]–[141]: Results contextualized — concentration-dependent gradient consistent with LAMP trial findings; compared with existing meta-analyses; clinical implications discussed (0.01% as preferred starting concentration).

Section and Topic	Item #	Checklist item	Location where item is reported
	23b	Discuss any limitations of the evidence included in the review.	Discussion, line [141]: Limitations: (1) only 2-year data for 0.05%; (2) premyopia evidence inconclusive; (3) long-term data limited to LAMP (no concurrent placebo post-Year 1); (4) mostly Asian populations; (5) limited studies precluded formal publication bias tests.
	23c	Discuss any limitations of the review processes used.	Discussion, line [141]: Limitations of review processes: pre-registered protocol (PROSPERO), PRISMA methodology, but limited to RCTs with extractable numerical data.
	23d	Discuss implications of the results for practice, policy, and future research.	Discussion, lines [140]–[143]: Clinical recommendations — 0.01% as preferred starting concentration; 0.025% or 0.05% for rapid progression; individualized treatment decisions. Future research: ≥3-year RCTs, standardized outcomes, racially diverse populations.
**OTHER INFORMATION**			
Registration and protocol	24a	Provide registration information for the review, including register name and registration number, or state that the review was not registered.	Methods, line [18]: "Pre-registered PROSPERO:

Section and Topic	Item #	Checklist item	Location where item is reported
			CRD420261366209"
	24b	Indicate where the review protocol can be accessed, or state that a protocol was not prepared.	Methods, line [18]: Protocol registered on PROSPERO (CRD420261366209).
	24c	Describe and explain any amendments to information provided at registration or in the protocol.	Methods: No amendments to protocol reported in the manuscript.
Support	25	Describe sources of financial or non-financial support for the review, and the role of the funders or sponsors in the review.	Manuscript, line [147]: "No funding or sponsorship was received for this study or publication of this article"
Competing interests	26	Declare any competing interests of review authors.	Manuscript, line [153]: "The authors have no conflicts of interest relevant to the content of this article to disclose."
Availability of data, code and other materials	27	Report which of the following are publicly available and where they can be found: template data collection forms; data extracted from included studies; data used for all analyses; analytic code; any other materials used in the review.	Manuscript, lines [150]–[151]: "All data generated or analyzed during this systematic review and meta-analysis are included in this published article and its supplementary materials." R scripts and extracted data available from corresponding authors upon reasonable request.

From: Page MJ, McKenzie JE, Bossuyt PM, Boutron I, Hoffmann TC, Mulrow CD, et al. The PRISMA 2020 statement: an updated guideline for reporting systematic reviews. BMJ 2021;372:n71. doi: 10.1136/bmj.n71. This work is licensed under CC BY 4.0. To view a copy of this license, visit https://creativecommons.org/licenses/by/4.0/
